# Autophagy regulates the localization and degradation of p16^INK4a^

**DOI:** 10.1111/acel.13171

**Published:** 2020-07-13

**Authors:** Philip R. Coryell, Supreet K. Goraya, Katherine A. Griffin, Margaret A. Redick, Samuel R. Sisk, Jeremy E. Purvis

**Affiliations:** ^1^ Department of Genetics University of North Carolina at Chapel Hill Chapel Hill North Carolina USA; ^2^ Curriculum for Bioinformatics and Computational Biology University of North Carolina at Chapel Hill Chapel Hill North Carolina USA; ^3^ Lineberger Comprehensive Cancer Center University of North Carolina at Chapel Hill Chapel Hill North Carolina USA; ^4^ Computational Medicine Program University of North Carolina at Chapel Hill Chapel Hill North Carolina USA

**Keywords:** autophagy, Ink4a, lysosomes, p16

## Abstract

The tumor suppressor protein p16^INK4a^ (p16) is a well‐established hallmark of aging that induces cellular senescence in response to stress. Previous studies have focused primarily on p16 regulation at the transcriptional level; comparatively little is known about the protein's intracellular localization and degradation. The autophagy–lysosomal pathway has been implicated in the subcellular trafficking and turnover of various stress‐response proteins and has also been shown to attenuate age‐related pathologies, but it is unclear whether p16 is involved in this pathway. Here, we investigate the role of autophagy, vesicular trafficking, and lysosomal degradation on p16 expression and localization in human epithelial cells. Time‐lapse fluorescence microscopy using an endogenous p16‐mCherry reporter revealed that serum starvation, etoposide, and hydrogen peroxide stimulate autophagy and drive p16 recruitment to acidic cytoplasmic vesicles within 4 hr. Blocking lysosomal proteases with leupeptin and ammonium chloride resulted in the accumulation of p16 within lysosomes and increased total p16 levels suggesting that p16 is degraded by this pathway. Furthermore, autophagy blockers chloroquine and bafilomycin A1 caused p16 aggregation within stalled vesicles containing autophagosome marker LC3. Increase of p16 within these vesicles coincided with the accumulation of LC3‐II. Knockdown of autophagosome chaperone p62 attenuated the formation of p16 aggregates in lysosomes, suggesting that p16 is targeted to these vesicles by p62. Taken together, these results implicate the autophagy pathway as a novel regulator of p16 degradation and localization, which could play a role in the etiology of cancer and age‐related diseases.

## Graphical abstract

Serum starvation, oxidative stress, or DNA damage activates autophagy and drives p62‐mediated recruitment of the senescence and aging biomarker p16 to lysosomes, where it is degraded. Autophagy inhibitors bafilomycin, chloroquine, leupeptin, and NH4Cl cause p16 aggregation within lysosomes and autophagosomes and raise total cellular p16 levels. These findings open up a novel thread of research into how p16 transport may influence cellular aging and tumor suppression.

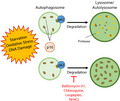

## 1 INTRODUCTION

The tumor suppressor protein p16^INK4a^ (CDKN2A, p16) is a member of the INK4 family of cyclin‐dependent kinase inhibitors, which play a critical role in cell‐cycle regulation. Expression of p16 prevents cellular proliferation by binding and inhibiting cyclin‐dependent kinases 4 and 6 (CDK4/6). In response to oncogene expression and prolonged DNA damage, p16 induces cellular senescence (permanent cell‐cycle arrest) (Serrano, 1997). As an organism ages, p16 accumulates in tissues, which triggers cellular senescence. Clearance of p16 expressing senescent cells has been linked to an increase in lifespan and a decrease in tumorigenesis (Baker et al., 2011). The correlation between p16 expression and aging is so strong that p16 is commonly used as a biomarker for aging (Krishnamurthy et al., 2004). While the mechanisms regulating transcription of p16 have been well described, studies about the localization and degradation of the p16 protein are lacking.

p16 is expressed in both the nucleus and the cytoplasm (Nilsson & Landberg, 2006); (Lu et al., 2014). Whereas the role of p16 in the nucleus as an inhibitor of CDK4/6 is well understood, its subcellular localization and function in the cytoplasm remains mysterious. Immunohistological studies of patient tumors have suggested p16 localization as a possible indicator of clinical prognosis. However, many of these studies present contradictory claims that indicate a complex role for p16 localization in tumor progression. For example, cytoplasmic p16 has been reported to be a predictor of poor prognosis in patients with astrocytic brain tumors (Arifin et al., 2006). However, cytoplasmic p16 has also been reported as correlating with the absence of metastasis in other cancer types, such as melanoma (Mihic‐Probst et al., 2006). Commonly used chemotherapeutic drugs, such as etoposide, can induce senescence (Petrova, Velichko, Razin, & Kantidze, 2016), but whether and to what extent these agents affect p16 localization has not been fully explored. Interestingly, p16 does not have a known nuclear localization signal (NLS) or a nuclear export signal (NES) (Dok, Asbagh, Van Limbergen, Sablina, & Nuyts, 2016), suggesting that an indirect mechanism of intracellular transport is responsible for shuttling p16 between different cellular compartments (Hu, Dammer, Ren, & Wang, 2015).

One potential mechanism for regulation of p16 localization is vesicular trafficking via the lysosomal endomembrane system. Lysosomes are cytoplasmic organelles involved in autophagy‐mediated protein degradation. Like p16, lysosomes are involved in senescence‐associated signaling pathways, and lysosome dysfunction has been linked to a myriad of age‐related pathologies and a decrease in lifespan (Carmona‐Gutierrez, Hughes, Madeo, & Ruckenstuhl, 2016); (Lee et al., 2006); (Platt, Boland, & van der Spoel, 2012). Similarly, lysosomes have also been targeted for lifespan extension therapies, such as intervention with rapamycin (Carmona‐Gutierrez et al., 2016). Recent studies have expanded beyond protein degradation and explored the role of lysosomes in subcellular localization of stress‐response proteins and the regulation of cell fate. For example, the mechanistic target of rapamycin (mTOR) was found to not only be recruited and degraded by lysosomes, but also plays an important role in lysosome formation and regulation of the entire autophagy pathway (Hu et al., 2016). Given the correlation of both autophagy and p16 expression with cellular aging and senescence, an intriguing hypothesis is that p16 localization, degradation, and regulation may be mediated by lysosomes and other members of this pathway. Previous experiments have shown that p16 can be degraded by the proteasome (Ben‐Saadon et al., 2004); however, no literature exists to support whether regulation can also occur through other known degradation mechanisms such as the autophagy/lysosomal pathway.

**FIGURE 1:**
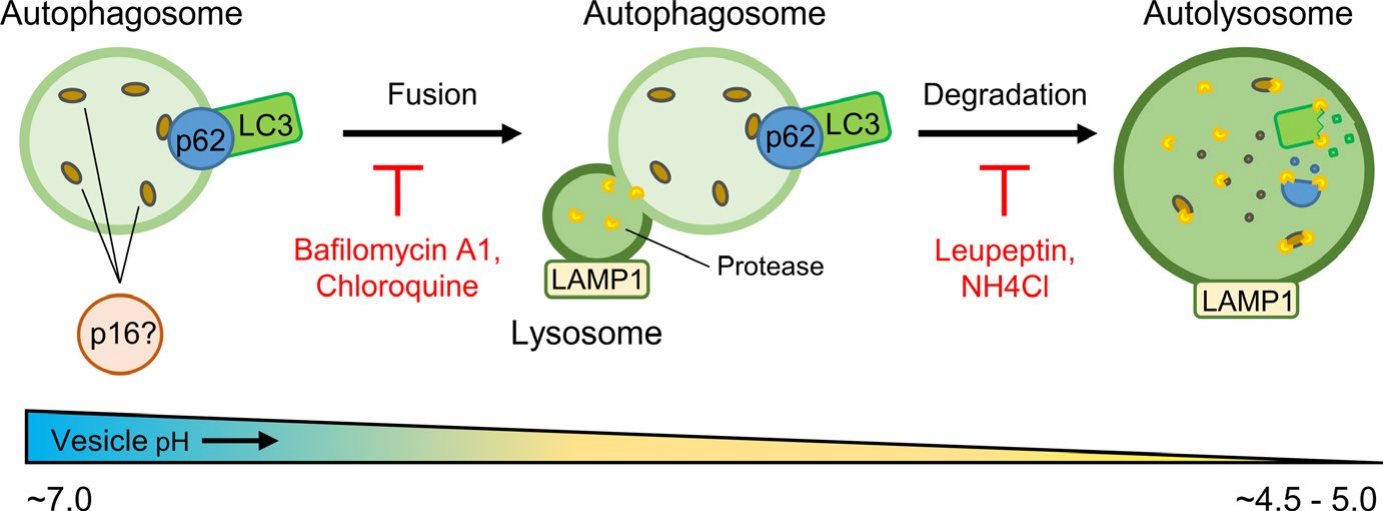
Autophagy pathway model depicting molecular markers and inhibitors. Autophagy chaperone protein p62 targets proteins destined for lysosome‐mediated degradation to autophagosomes, which are identifiable by autophagosome membrane marker LC3. Autophagosomes fuse to lysosomes (identifiable by lysosome membrane marker LAMP1) containing low‐pH‐dependent proteases, forming autolysosomes. The pH of these vesicles lowers throughout this process, provoking the degradation of proteins within the autolysosome, including p62 and LC3. Autophagy inhibitors bafilomycin A1 and chloroquine act by preventing the fusion of autophagosomes and lysosomes. Leupeptin inhibits protease within lysosomes and autolysosomes. Ammonium chloride (NH4CI) prevents lysosomal protease activity by raising vesicular pH

As shown in Figure 1, the autophagy pathway consists of several sequential steps, beginning with stimulation by nutrient starvation or cellular stress, followed by interaction between autophagosomes and lysosomes, and ending with the lysosomal degradation of proteins. Ubiquitinated proteins or protein aggregates can be targeted for lysosomal degradation by ubiquitin‐binding protein p62 (also known as sequestosome 1; SQSTM1). p62‐bound proteins are enveloped by autophagosomes, which are identifiable by autophagosome marker membrane‐bound microtubule‐associated protein 1A/1B‐light chain 3 (LC3). Autophagosomes then fuse to lysosomes (identifiable by lysosomal‐associated membrane protein 1; LAMP1) containing low‐pH‐dependent hydrolases, forming autolysosomes. The pH of these vesicles lowers throughout this process, provoking the degradation of proteins within the autolysosome, including p62 and LC3. Autophagic flux, or the rate at which proteins are degraded by this pathway, can change in response to cellular stress and nutrient availability. Moreover, changes in autophagic flux can rapidly affect the localization and expression of proteins involved in this pathway (Loos, du Toit, & Hofmeyr, 2014). Accordingly, measurements of autophagy often require the use of inhibitors to capture proteins in transit within this pathway. Examples of well‐characterized autophagy inhibitors include leupeptin, a selective lysosomal protease inhibitor, ammonium chloride (NH4Cl), which raises vesicular pH, and bafilomycin A1 and chloroquine, which act by preventing the fusion of autophagosomes and lysosomes (Yang et al., 2013).

In this study, we investigated the relationship between p16 and the autophagy/lysosomal pathway in human cells. To do this, we subjected human retinal pigment epithelial (RPE‐1) cells to three cellular stresses that have previously been shown to induce both autophagy and cell‐cycle arrest: nutrient deprivation via serum starvation, oxidative stress via hydrogen peroxide, and genotoxic stress via the chemotherapeutic drug etoposide (Katayama, Kawaguchi, Berger, & Pieper, 2007). By engineering a live‐cell reporter for p16, we found that activation of autophagy caused p16 to accumulate in acidic cytoplasmic vesicles within 24 hr. RPE‐1 cells treated with lysosomal protease inhibitors leupeptin and NH4Cl displayed strong colocalization between p16 and lysosomes and increased total p16 levels. Furthermore, blocking autophagosome‐to‐lysosome fusion led to increased levels of p16 within LC3‐positive vesicles. Knockdown of autophagosome chaperone protein p62 diminished the ability of p16 to aggregate and colocalize with lysosomes. Taken together, these results show that p16 is localized and degraded through the autophagy/lysosomal pathway, implicating the autophagy pathway as a regulator of p16 and senescence.

## 2 RESULTS

### 2.1 Autophagy recruits p16 to acidic organelles

Autophagy is a highly dynamic process involving rapid protein transport and turnover known as autophagic flux. As a consequence, many autophagy markers and proteins targeted for degradation are difficult to measure (Loos et al., 2014; Yoshii & Mizushima, 2017). Immunostaining of fixed cells can capture protein localization only at a single point in time. Furthermore, permeabilization using harsh detergents can destroy membrane‐bound organelles such as endosomes, lysosomes, and autophagosomes (Goldenthal, Hedman, Chen, August, & Willingham, 1985). As an alternative approach, fluorescently tagged protein reporters have been employed to accurately visualize and track temporal changes of members of the autophagy pathway and proteins destined to this pathway for degradation (Loos et al., 2014; Yoshii & Mizushima, 2017).

To monitor p16 protein expression and localization in real time, we developed a live‐cell reporter. A fluorescent p16‐mCherry fusion protein was incorporated at the endogenous p16 locus in RPE‐1 cells using CRISPR‐mediated homologous recombination (Figure S1a–g). mCherry was selected because of its pH stability and ability to maintain fluorescence under acidic conditions, including within the lysosomal lumen (Bjørkøy et al., 2009; Shaner, Steinbach, & Tsien, 2005). This resulted in the creation of an endogenously‐tagged p16‐mCherry fusion reporter cell line (henceforth referred to as RPE p16‐mCherry) to measure vesicular p16 during live‐cell fluorescence experiments.

**FIGURE 2:**
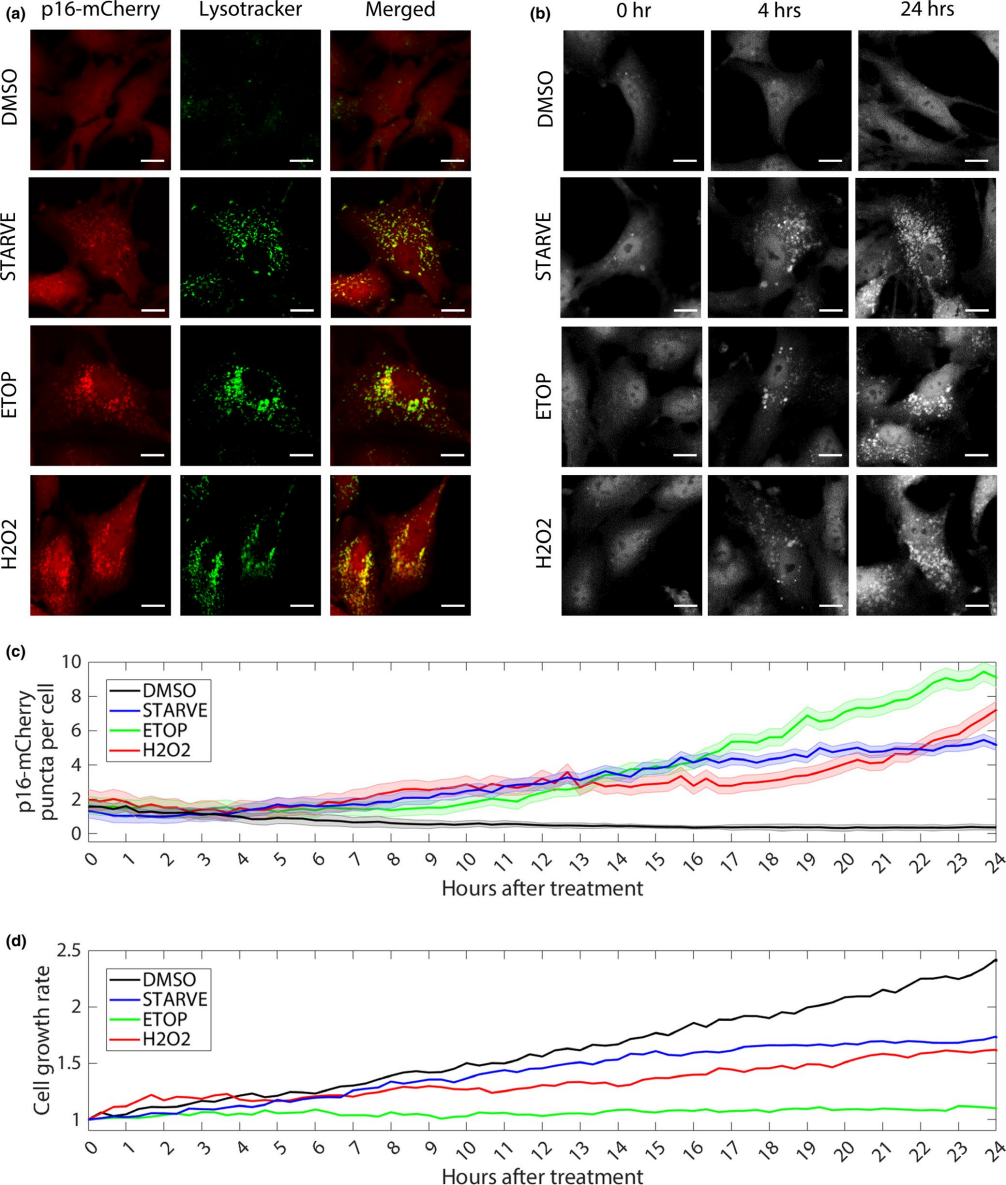
Dynamics of p16 localization in response to autophagy stimulation. RPE p16‐mCherry cells were treated with DMSO, etoposide (20 μM), H2O2 (200 μM), or serum starved for 24 hr. (a) Fluorescent p16‐mCherry shown in red; LysoTracker staining of acidic organelles shown in green. Scale bars = 10 μM. (b) Time‐lapse images of p16‐mCherry cells after treatments. (c) Quantification of time‐lapse images showing total mean per frame of mean p16‐mCherry puncta per cell. 1 hr = 3 frames. *n* > 200 cells per frame for each condition. Shading represents standard error of the mean. (d) Relative cell growth rate for each condition quantified by dividing the total number of cells per frame by the total number of cells in frame 1

We first asked how p16 expression and localization changes in response to autophagy. To do this, we subjected RPE p16‐mCherry cells to serum starvation, hydrogen peroxide, or etoposide treatment. To monitor the activation of autophagy, cells were also treated with LysoTracker, a live‐cell chemical stain for V‐ATPase activity in acidified vesicles. After 24 hr, DMSO‐treated control cells exhibited sparse LysoTracker staining, as well as diffuse cytoplasmic p16‐mCherry, demonstrating that p16 is expressed and autophagy is inactive under basal conditions (Figure 2a). In contrast, serum starvation, hydrogen peroxide, and etoposide induced bright cytoplasmic puncta in response to LysoTracker staining, demonstrating that these treatments were sufficient to trigger autophagy. Moreover, these cells accumulated cytoplasmic p16‐mCherry puncta that colocalized with LysoTracker, demonstrating that p16‐mCherry localizes to acidic cytoplasmic compartments in response to these stresses. Time‐lapse images revealed that p16‐mCherry puncta began forming 4 hr after treatment and increased over the course of 24 hr (Figure 2b and c). Furthermore, growth‐curve analysis revealed all three treatments that induced p16‐mCherry puncta were also sufficient to induce cell‐cycle arrest (Figure 2d).

Together, these data suggest that cellular stress induced by nutrient starvation, oxidative damage, or DNA damage halts the cell‐cycle, induces autophagy, and sequesters p16 to acidic compartments in the cytoplasm. Recruitment of p16 to these compartments occurred within 4 hr after exposure to these stresses and continued for at least 24 hr. These results implicate the autophagy pathway as a regulator of p16 protein localization.

### 2.2 Blocking lysosomal degradation causes p16 aggregation within lysosomes

We next asked how disruption of autophagy affects the expression and subcellular localization dynamics of p16. The autophagy‐mediated protein degradation pathway involves the acidification of lysosomes in order to activate low‐pH‐dependent proteases within the lysosomal lumen. Our live‐cell experiments revealed colocalization between p16‐mCherry and acidic organelles, which may be lysosomes. In order to confirm this finding in a nonreporter cell line, we tested whether blocking lysosomal degradation in unmodified RPE‐1 cells while stimulating autophagy resulted in the accumulation of p16 within lysosomes.

Autophagy stimulates the conversion of LC3‐I to LC3‐II, which is subsequently degraded within lysosomes. Accordingly, disrupting autophagy results in the accumulation of LC3‐II within cells. Autophagy can be blocked by exposing cells to NH4Cl, which prevents the acidification of lysosomes, and leupeptin, a selective lysosomal protease inhibitor (Yang et al., 2013). Protein analysis via immunoblot demonstrated that 24‐hr exposure to leupeptin combined with NH4Cl significantly increased LC3‐II levels, confirming that lysosomal degradation was sufficiently blocked by this treatment (Figures S2a and b). Furthermore, blocking lysosomes induced greater LC3‐II accumulation in cells when autophagy was stimulated by serum starvation, hydrogen peroxide, or etoposide, relative to cells that were unstimulated.

**FIGURE 3:**
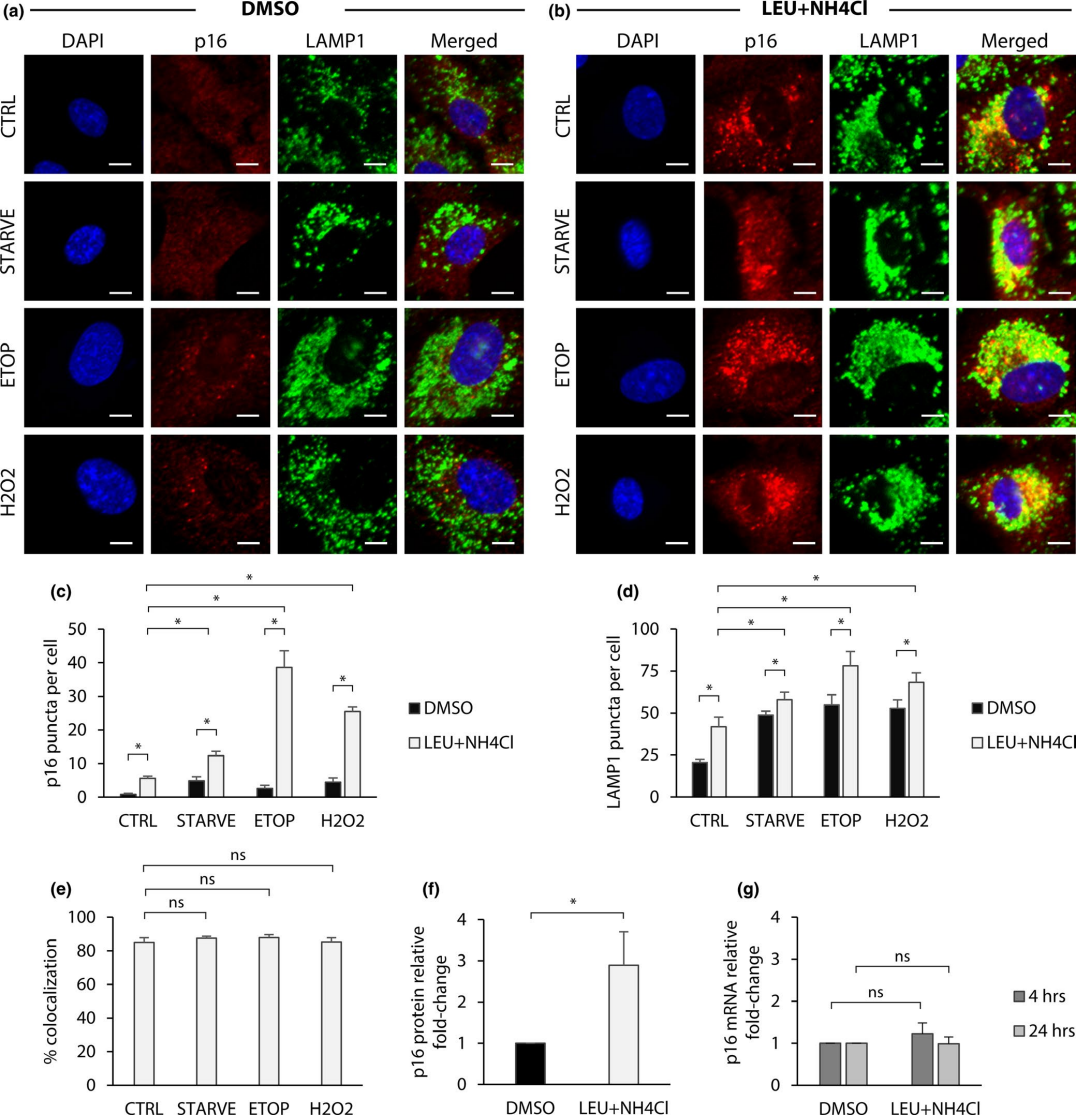
Autophagy recruits p16 to lysosomes. RPE‐1 cells were treated with etoposide (20 μM), H2O2 (200 μM), or serum starved for 24 hr. Additionally, each group was treated with DMSO or leupeptin (100 μM) and NH4CI (10 mM). Cells were then fixed and permeabilized with digitonin for immunofluorescence staining. (a) Cells treated with DMSO, in addition to the treatments previously described. DAPI shown in blue; p16 shown in red; and LAMP1 shown in green. Scale bars = 10 μM. (b) Cells treated with leupeptin + NH4CI, in addition to the treatments previously described. (c) Quantification of p16 puncta per cell. (d) Quantification of LAMP1 puncta per cell. (e) Quantification of % colocalization for LEU + NH4CI treatment groups, calculated as the percent of total p16 puncta per cell colocalized with LAMP1 puncta. For c‐e, results are the mean of sample means obtained from nine images per group with at least 100 cells per image. Statistical significance determined by two‐way ANOVA and Bonferroni correction (*n* = 9). ns = *p*>.01 and * = *p* < .01. (f) Quantification of western blots for cells treated with leupeptin (100 μM) and NH4CI (10 mM) for 24 hr. Proteins normalized to actin. Statistical significance determined by one‐way ANOVA (*n* = 3).* = *p* < .05. (g) RT‐qPCR for cells treated with leupeptin (100 μM) and NH4CI (10 mM) for 4 or 24 hr. Transcripts normalized to actin. Statistical significance determined by two‐way ANOVA (*n* = 3). Ns = *p*>.05. All error bars = standard deviation

To test whether p16 is recruited to lysosomes we performed immunofluorescence staining and quantified the amount of p16 colocalizing with lysosomes per cell (Materials and Methods). This protocol was specifically designed to avoid the destruction of membrane‐bound organelles by permeabilizing fixed cells with digitonin, a selective detergent that punctures the plasma membrane while leaving endomembrane vesicles intact (Jaattela & Nylandsted, 2015). Using leupeptin + NH4Cl, we blocked lysosomal degradation while stimulating autophagy via serum starvation, hydrogen peroxide, or etoposide treatment. For all treatments, stimulation of autophagy in cells with active lysosomes produced few cytoplasmic p16 puncta (Figure 3a and c). However, blocking lysosomal degradation with leupeptin and NH4Cl for 24 hr resulted in the accumulation of cytoplasmic p16 puncta that colocalized with LAMP1, suggesting that a proportion of p16 was recruited to lysosomes (Figure 3b and c). Stimulation of autophagy significantly increased the total number of p16 puncta per cell when lysosomal degradation was inhibited. Additionally, autophagy greatly increased the number of LAMP1 puncta per cell, suggesting an upregulation in lysosome production (Figure 3d). Although simultaneous autophagy activation and blocking increased the number of lysosomes per cell relative to blocking alone, the percentage of p16 puncta colocalized with lysosomes was not significantly changed, suggesting that most p16 aggregates that form were inside lysosomes (Figure 3e).

Intriguingly, the number of p16‐positive lysosomes in unstimulated cells was significantly increased by blocking autophagy, suggesting that p16 in RPE‐1 cells is always in autophagic flux. To test this, we performed protein analysis by western blot on cells treated with leupeptin and NH4Cl. Blocking lysosomal degradation for 24 hr increased total p16 protein levels (Figure 3f and Figure S2d). Additionally, RT‐qPCR performed 4 and 24 hr after treatment revealed that increased p16 protein in response to lysosome inhibition was not the result of de novo p16 transcription (Figure 3g). Together, these results confirm that p16 can be degraded by lysosomes and is always in autophagic flux in RPE‐1 cells. Furthermore, stimulation of autophagy by serum starvation, hydrogen peroxide, or etoposide enhances p16 localization to lysosomes, and cells accumulate lysosomal p16 when autophagic degradation is disrupted.

### 2.3 Disrupting autophagosome–lysosome fusion causes p16 aggregation within autophagosomes

Autophagosomes are endomembrane vesicles that accumulate cargo destined for autophagy‐mediated destruction. Lysosomes fuse to autophagosomes, forming autolysosomes, in which autophagosome‐associated proteins and the content within them are degraded. Bafilomycin A1 and chloroquine are potent inhibitors of late‐stage autophagy that act by preventing fusion between autophagosomes and lysosomes (Yamamoto et al., 1998); (Zhang, Qi, Wu, & Qin, 2013). Accordingly, autophagosome membrane marker LC3 and other proteins destined for lysosomal degradation accumulate within stalled autophagosomes (Bjørkøy et al., 2009). In Figure 3, we demonstrated that p16 localizes to lysosomes when autophagy is stimulated by nutrient deprivation or cellular stress. We therefore asked whether p16 is targeted to lysosomes by autophagosomes in response to autophagy.

To test this, we first exposed RPE‐1 cells to bafilomycin or chloroquine for 24 hr to test if autophagy was blocked by these treatments. Protein analysis via immunoblot revealed a significant increase in LC3‐II, which confirmed that autophagy was sufficiently blocked (Figure S2a and b). Furthermore, bafilomycin and chloroquine treated cells had greater LC3‐II accumulation when autophagy was stimulated by serum starvation, hydrogen peroxide, or etoposide, relative to cells that were unstimulated.

**FIGURE 4:**
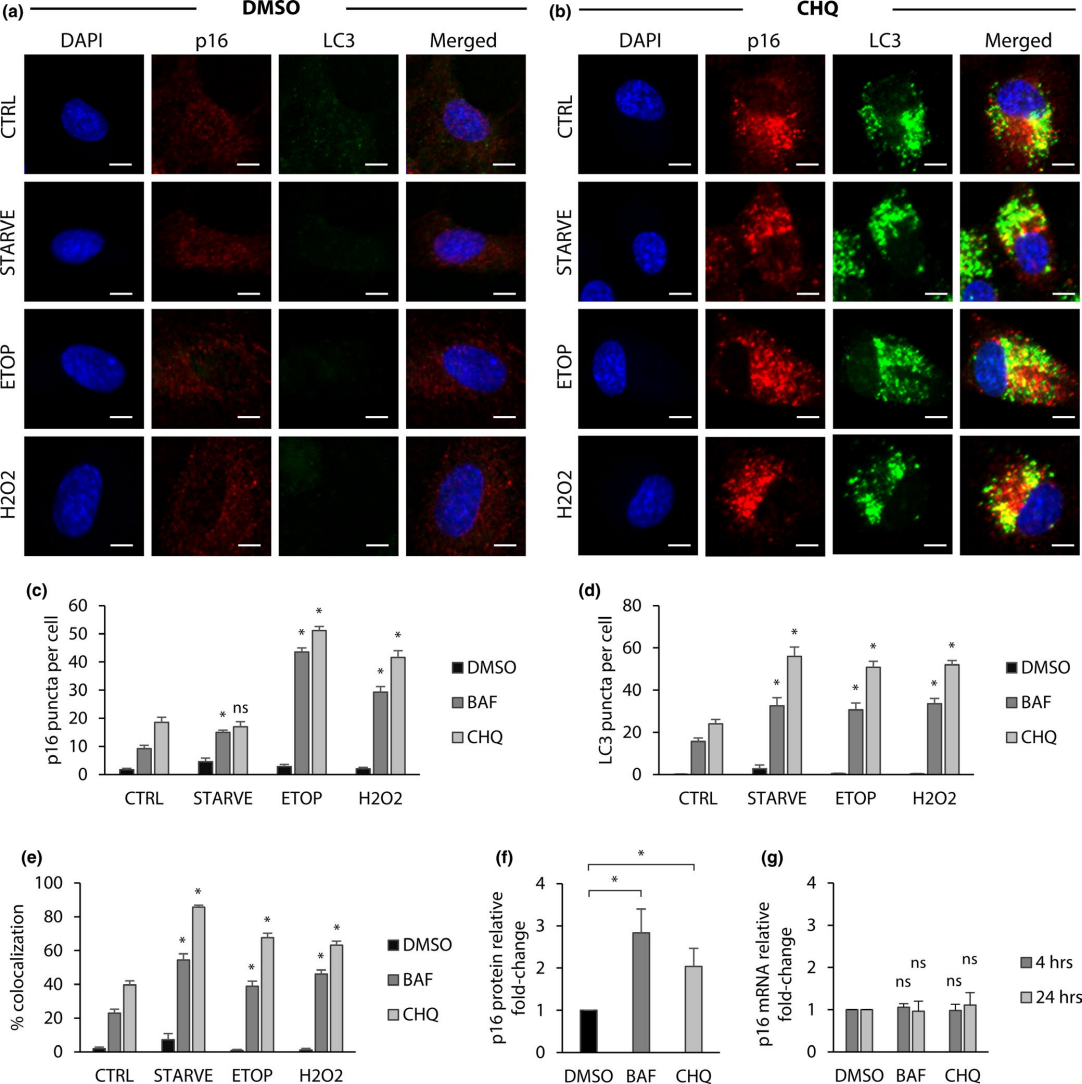
Blocking autophagy results in p16 accumulation within autophagosomes. RPE‐1 cells were treated with etoposide (20 μM), H2O2 (200 μM), or serum starved for 24 hr. Additionally, each group was treated with DMSO, bafilomycin (100 nM), or chloroquine (40 μM). Cells were then fixed and permeabilized with digitonin for immunofluorescence staining. (a) Cells treated with DMSO, in addition to the treatments previously described. DAPI shown in blue; p16 shown in red; and LC3 shown in green. Scale bars = 10 μM. (b) Cells treated with chloroquine, in addition to the treatments previously described. (c) Quantification of p16 puncta per cell. (d) Quantification of LC3 puncta per cell. (e) Quantification of % colocalization, calculated as the percent of total p16 puncta per cell colocalized with LC3 puncta. For c‐e, results are the mean of sample means obtained from 10 images per group with at least 100 cells per image. Statistical significance determined by two‐way ANOVA and Bonferroni correction (*n* = 10). ns = *p *> .01 and* = *p* < .01 relative to respective CTRL. (f) Quantification of western blots for cells treated with bafilomycin (100 nM) or chloroquine (40 μM) for 24 hr. Proteins normalized to actin. Statistical significance determined by one‐way ANOVA (*n* = 3). * = *p* < .05. (g) RT‐qPCR for cells treated with bafilomycin (100 nM) or chloroquine (40 μM) for 4 or 24 hr. Transcripts normalized to actin. Statistical significance determined by two‐way ANOVA (*n* = 3). ns = *p *> .05 relative to respective DMSO. All errors bars = standard deviation

Next, we tested whether stimulating autophagy while blocking autophagosome–lysosome fusion via bafilomycin or chloroquine resulted in the accumulation of p16 within autophagosomes. Immunofluorescence revealed that cytoplasmic p16 in control cells was diffuse, and autophagosome marker LC3 was either sparse or undetectable, a phenomenon known to be caused by rapid autophagic flux (Yoshii & Mizushima, 2017) (Figure 4a and c). However, disruption of autophagy via chloroquine or bafilomycin treatment caused aggregation of cytoplasmic p16 puncta, which colocalized with LC3‐positive puncta, indicating that p16 was accumulated within autophagosomes (Figure 4b,c, and Figure S2c). When autophagy was blocked, the number of LC3 puncta was significantly increased in cells exposed to serum starvation, hydrogen peroxide, or etoposide, suggesting that autophagosome production was amplified by autophagy stimulation (Figure 4d). These treatments also significantly increased both the number of p16 puncta per cell and their colocalization with LC3 puncta, demonstrating that stimulation of autophagy drives p16 recruitment to autophagosomes (Figure 4e). To confirm that these cytoplasmic puncta were p16, and to test whether p16 itself affects autophagosome formation, we silenced p16 via siRNA and performed immunostaining to detect p16 and LC3 accumulation in cells treated with chloroquine. Knockdown of p16 eliminated p16 puncta without disrupting LC3 expression and formation in response to chloroquine, demonstrating that p16 is not required for autophagosome formation (Figures S3a–c).

Finally, to test whether p16 protein accumulates in cells with dysfunctional autophagy we treated RPE‐1 cells with chloroquine or bafilomycin and performed protein analysis via western blot. Both chloroquine and bafilomycin were sufficient to increase total p16 protein levels after 24 hr (Figure 4f and Figure S2d). Additionally, RT‐qPCR performed 4 and 24 hr after both treatments revealed that increased p16 protein in response to blocking autophagosome–lysosome fusion was not the result of de novo p16 transcription (Figure 4g). Together, these results validate that p16 is shuttled through the autophagy pathway by autophagosomes, which implicates this pathway as a potential regulator of p16 and senescence.

### 2.4 Autophagosome chaperone p62/SQSTM1 mediates p16 recruitment to lysosomes

Autophagy can be selective for certain proteins and macromolecules targeted for degradation. For selective autophagy, ubiquitinated proteins or protein aggregates can be targeted to autophagosomes by ubiquitin‐binding protein p62/SQSTM1. Although p16 does not contain a lysine residue, N‐terminal ubiquitination of p16 has been reported (Ben‐Saadon et al., 2004). Therefore, we tested whether p16 is selectively targeted to the autophagy pathway via p62 by studying p16 localization in response to the silencing of p62 by siRNA.

First, we tested whether p62 knockdown inhibited the autophagy pathway by silencing p62 and studying its effects on LC3 puncta formation. To do this, we co‐treated RPE‐1 cells with either siRNA targeting p62 or control scramble siRNA, as well as DMSO or the autophagy blocker chloroquine for 24 hr. Immunofluorescence microscopy and protein analysis via western blot confirmed robust knockdown of p62 24 hr after siRNA treatment (Figures S3d–g). Additionally, cytoplasmic LC3 and p62 puncta were present 24 hr after exposing cells to chloroquine and scramble siRNA, suggesting that these proteins accumulated within stalled autophagosomes. Knockdown of p62 alone was not sufficient to induce LC3 puncta. Additionally, p62 knockdown ablated p62‐ but not LC3‐puncta formation in response to chloroquine, suggesting that silencing p62 does not block LC3 expression or autophagy.

**FIGURE 5:**
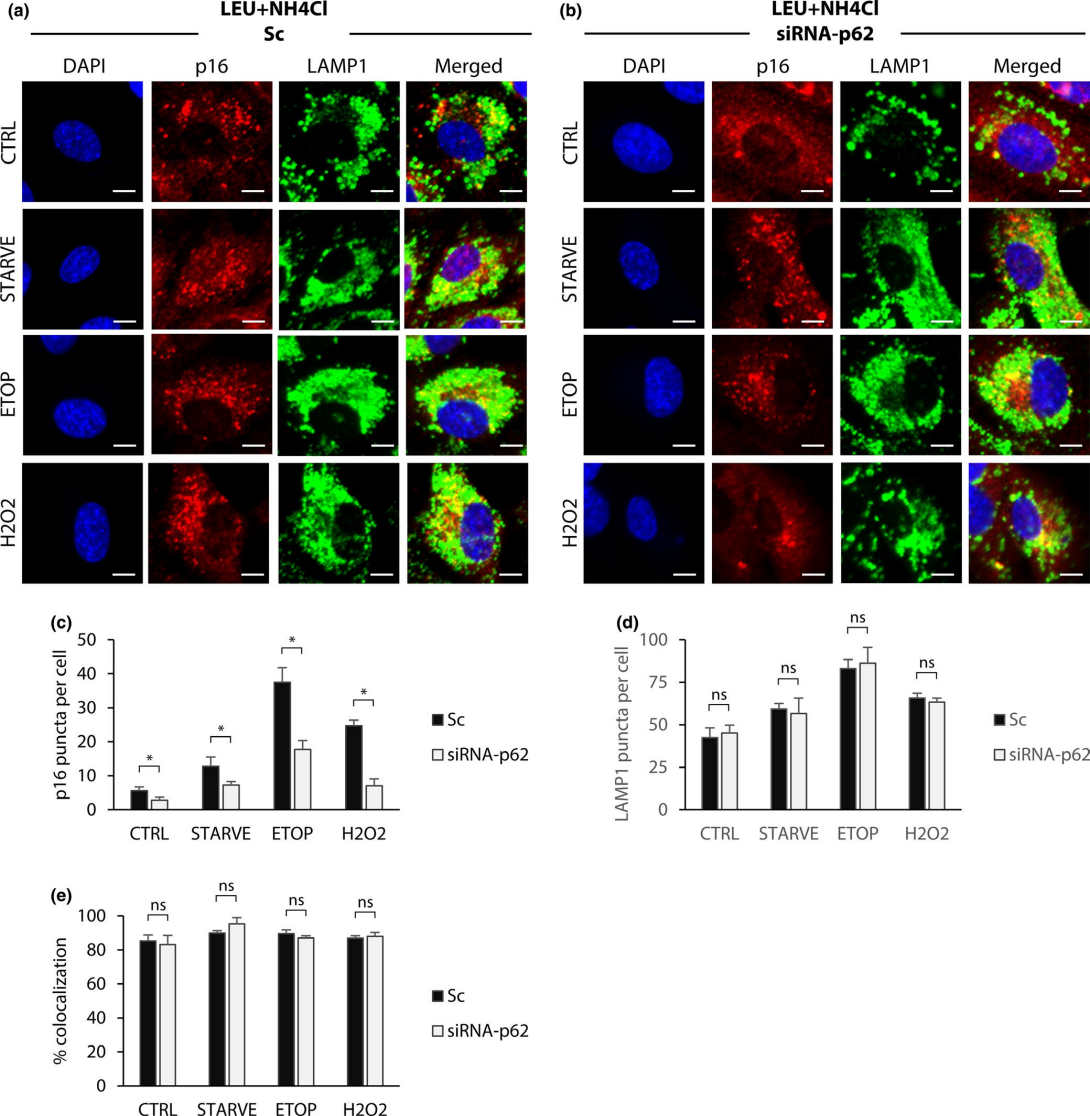
p16‐lysosome recruitment is mediated by p62. RPE‐1 cells transfected with siRNA‐p62 or scramble control (Sc) were treated with etoposide (20 μM), H2O2 (200 μM), or serum starved for 24 hr. Additionally, each group was treated with DMSO or leupeptin (100 μM) and NH4CI (10 mM). Cells were then fixed and permeabilized with digitonin for immunofluorescence staining. (a) Cells treated with leupeptin + NH4CI and scramble control, in addition to the treatments previously described. DAPI shown in blue; p16 shown in red; and LAMP1 shown in green. Scale bars = 10 μM. (b) Cells treated with leupeptin + NH4CI and siRNA targeting p62, in addition to the treatments previously described. (c) Quantification of p16 puncta per cell. (d) Quantification of LAMP1 puncta per cell. (e) Quantification of % colocalization, calculated as the percent of total p16 puncta per cell colocalized with LAMP1 puncta. For c‐e, results are the mean of sample means obtained from nine images per group with at least 100 cells per image. Statistical significance determined by two‐way ANOVA and Bonferroni correction (*n* = 9). ns = *p*>.01 and* = *p* < .01. All error bars = standard deviation

Next, we tested whether p62 knockdown affects p16 recruitment to lysosomes. To do this, we repeated the experiment shown in Figure 3 by stimulating autophagy while inhibiting lysosomal degradation with leupeptin + NH4Cl in cells with silenced p62. Immunostaining for p16 and lysosome marker LAMP1 revealed that silencing of p62 resulted in the formation of fewer cytoplasmic p16 aggregates in response to autophagy stimulation and blocking lysosomal degradation compared to scramble‐treated control cells (Figure 5a–c). The number of LAMP1 puncta per cell was not significantly affected by p62 knockdown, suggesting that the loss of p16 aggregates was not caused by disruption of lysosome formation (Figure 5d). Of the fewer p16 puncta that did form, knockdown of p62 did not affect the percentage of those puncta localized to lysosomes, indicating that p16 was reaching lysosomes through additional pathways other than p62‐mediated chaperoning (Figure 5e). Taken together, these results demonstrate that autophagy triggers recruitment of a proportion of p16 to lysosomes in a manner that is dependent on the expression of the chaperone p62.

## 3 DISCUSSION

In summary, our study demonstrates that localization and degradation of the p16 protein is regulated in part by the autophagy–lysosomal pathway in human RPE‐1 cells. Live‐cell experiments using a p16‐mCherry fluorescent reporter revealed that autophagy stimulation induces lysosomal p16 enrichment within 4 hr and can be triggered by serum starvation, oxidative stress by hydrogen peroxide, and genotoxic stress by the chemotherapeutic agent etoposide. Blocking autophagy using leupeptin, chloroquine, or bafilomycin greatly increases the amount of p16 inside lysosomes, demonstrating that p16 can be degraded by this pathway. Additionally, we found that p16 is recruited to lysosomes by the chaperone protein p62. Together, these results reveal an unappreciated mode of regulation of the p16 protein in human cells.

Traditionally, protein localization has been studied with immunohistological experiments using antibodies targeting the protein of interest. However, these methods require the fixation of cells, which prevents temporal analysis of protein expression and localization. The autophagy pathway and endomembrane system is dynamic, mobile, and known to induce drastic changes in protein localization in a relatively short time‐frame. By creating an endogenous p16‐mCherry reporter in human cells, we have contributed a novel tool for examining p16 expression and localization over time. Use of this reporter in future experiments will help to further our understanding of p16 dynamics in response to a multitude of chemotherapeutic agents, cellular stresses, and inducers of autophagy dysfunction.

Further study is required to identify the precise mechanisms that control p16 localization. For example, it is not known which domains on the p16 protein are responsible for autophagosomal and lysosomal recruitment. While we have found that p62 promotes p16 recruitment to lysosomes, the endomembrane‐transport system is complex, with many additional chaperone proteins and post‐translational modifiers involved in recruiting, sorting, and shuttling cargo between different compartments of the cell. Determining the specific factors that control p16 transport could reveal potential drug targets for disease and anti‐aging therapies.

We found that stimulating autophagy in RPE‐1 cells while blocking lysosomal degradation led to a significant increase in lysosomal p16 aggregates. Interestingly, blocking autophagy also induced the formation of lysosomal p16 aggregates to some extent even when autophagy was not stimulated. This suggests that p16 is continually in autophagic flux in these cells, which may explain how proliferating cells can sustain basal levels of p16 expression without inducing cell‐cycle arrest. Additionally, we have demonstrated that activation of autophagy recruits p16 to lysosomes for degradation, which may prevent p16‐induced senescence despite increases in p16 expression stimulated by cellular stress. Our study expands this relationship to the tumor suppressor p16, and links p16 localization to lysosomal function, which both serve as key regulators of senescence, disease, and aging.

Since the p16 protein has long been known to promote cell‐cycle arrest through inhibition of CDK4/6 in the nucleus, these results suggest a potential competition between the autophagy and senescence pathways through the sequestration of p16. Under this hypothetical model, stress induces the production of p16, which is quickly recruited to autophagosomes and degraded by lysosomes via the autophagy pathway. Over time, either through enhanced transcriptional activity or through p16 protein localization outside of lysosomes, p16 is able to enter the nucleus to bind to CDK4/6 and arrest the cell cycle. However, if the autophagy pathway is inhibited, p16 degradation is perturbed, which could lead to premature senescence. From this model, we posit that autophagy “buys time” for cells undergoing stress to determine whether the damage is manageable and cells are able to resume proliferation once the stress conditions are eliminated. Alternatively, if stress conditions persist, or if autophagy is dysregulated, the cell enters senescence. Future studies will be necessary to determine whether sequestration of p16 through the autophagy–lysosomal pathways reduces a cell's tendency to undergo senescence.

Finally, the observation that p16 localizes to and is degraded by lysosomes represents a potentially novel thread of research for cancer cell biology. Commonly used chemotherapeutic drugs can induce increases in p16 expression in patients, but the effect of these agents on p16 localization in single cells has not been fully explored. Understanding how these therapies affect p16 localization could illuminate how these treatments work at a mechanistic level. The ability to control senescence and attenuate cell growth via combined treatment with chemotherapeutics and well‐established autophagy inhibitors could have major implications for cancer treatment. Beyond this application, the ability to slow or prevent senescence in healthy proliferating cells, such as stem cells, could lead to potential new therapies for other age‐related diseases. In addition, we believe it is worth exploring the role of p16 in lysosomal storage diseases, which account for dozens of disorders associated with the brain, skin, heart, and central nervous system.

## 4 MATERIALS AND METHODS

### 4.1 Cell culturing and maintenance

For routine maintenance and growth, RPE‐1 cells were maintained in culture medium consisting of DMEM (Gibco 11995–065) supplemented with 10% FBS (Millipore Sigma TMS‐013‐B). For live‐cell fluorescent microscopy experiments, RPE p16‐mCherry cells were maintained in culture medium consisting of FluoroBrite DMEM (Gibco A1896701) supplemented with 10% FBS and 1% L‐glutamine. All cell lines were maintained in an incubator at 37°C and 5% CO_2_.

### 4.2 Fluorescence microscope

All fluorescence microscopy experiments were performed using a Nikon Ti Eclipse microscope operated by NIS Elements software V4.60 with an Andor ZYLA 4.2 camera. For live‐cell experiments, cells were imaged while being maintained in custom stage enclosure at 37°C and 5% CO_2_.

### 4.3 Live‐cell experiments and fluorescence quantification

Retinal pigment epithelial p16‐mCherry reporter cells were plated onto glass‐bottom 6‐well plates (Cellvis P06‐1.5H‐N) in DMEM FluoroBrite culture medium. After 24 hr, media was replaced with DMEM FluoroBrite culture medium supplemented with either 20 µM etoposide (MedChemExpress HY‐13629), 200 µM hydrogen peroxide, 0.5% DMSO, or starvation medium (DMEM FluoroBrite with 0% FBS). Time‐lapse fluorescent microscopy was then performed for 24 hr at 20 min/frame intervals.

Background subtraction of images was performed by rolling ball subtraction in ImageJ. Segmentation, counting, and fluorescence quantification of cells and subcellular compartments were performed in CellProfiler.

### 4.4 Immunofluorescence, siRNA, and fluorescence quantification

Retinal pigment epithelial‐1 cells were plated at low density onto glass‐bottom 24‐well plates (Cellvis P24‐1.5H‐N) in culture medium and grown to 50% confluence. Media was then replaced with culture medium supplemented with either 20 µM etoposide, 200 µM hydrogen peroxide, 0.5% DMSO, or starvation medium (DMEM with 0% FBS). Depending on experimental conditions, cells were also treated with 100 μM leupeptin (MedChemExpress HY‐18234A) and 10 mM ammonium chloride (Sigma 254134), 40 μM chloroquine (MedChemExpress HY‐17589), or 100 nM bafilomycin A1 (MedChemExpress HY‐100558). Depending on experimental conditions, cells were also treated with Lipofectamine RNAiMAX Transfection Reagent (ThermoFisher 13778030) and siRNA‐p16 (Dharmacon), siRNA‐p62 (Dharmacon), or nontargeting scramble siRNA (Dharmacon D‐001206–13–05). 24 hr later, cells were washed with ice‐cold PBS supplemented with 40 mM NH4Cl to stop lysosomal protease activity and then fixed with PBS containing 3.4% paraformaldehyde and 0.1% glutaraldehyde for 5 min at room temperature. Cells were then permeabilized and blocked with 0.02% digitonin (Invitrogen BN2006) in LI‐COR Odyssey Blocking Buffer (927–40000) containing 5% serum for 30 min at room temperature. All following steps were performed in blocking buffer containing 0.02% digitonin and 5% serum. First, cells were incubated at room temperature for 1 hr in blocking buffer containing Anti‐CDKN2A/p16INK4a antibody (Abcam ab108349), Anti‐LAMP1 antibody (Abcam ab25630), Anti‐SQSTM1/p62 antibody (Abcam ab56416), or Anti‐LC3B antibody (Abcam ab192890). Cells were washed three times for 5 min with PBS and then incubated for 1 hr at room temperature in wash buffer containing mouse and rabbit conjugated secondary antibodies. For p62 and LC3 staining experiments, cells were then washed three times for 5 min with PBS, blocked again for 1 hour in wash buffer, and then incubated overnight at 4°C in wash buffer containing Anti‐CDKN2A/p16INK4a conjugated (Alexa Fluor 647) antibody (Abcam ab192054). Cells were then washed with PBS containing 1 µg/ml DAPI for 5 min at room temperature, followed by three washes with PBS before visualization.

Segmentation, counting, and fluorescence quantification of cells, subcellular compartments, and fluorescent puncta were performed in CellProfiler. Measured results were plotted and tested for statistical significance in MATLAB using ANOVA and Bonferroni correction for multiple comparison analysis.

### 4.5 Western blot and protein quantification

Retinal pigment epithelial‐1 cells used for protein analysis were plated at low density onto 6‐well cell plates (Eppendorf 30720113) in culture medium and treated for each experimental condition at 50% confluence. For whole‐cell protein analysis, cells were lysed with ice‐cold RIPA buffer containing protease and phosphatase inhibitors. Lysates were separated on a gradient gel (TGX, Bio‐Rad) and transferred to a PVDF membrane. Membranes were blocked with blocking buffer (LI‐COR Odyssey Blocking Buffer 927–40000) for 1 hr before probing with primary antibodies for p16 (Abcam ab108349), SQSTM1/p62 (Abcam ab56416), LC3B (Cell Signaling E5Q2K), and beta‐actin (Cell Signaling 8H10D10) in blocking buffer overnight at 4°C. Membranes were washed and probed with secondary antibodies (LI‐COR goat anti‐mouse IRDye800 and goat anti‐rabbit IRDye680) for 1 hr at room temperature and visualized using the LI‐COR Odyssey CLx Imaging System. Proteins were normalized to actin and quantified using ImageJ. Quantified results were tested for statistical significance in MATLAB using two‐way ANOVA and Bonferroni correction for multiple comparison analysis.

### 4.6 Quantitative PCR

Retinal pigment epithelial‐1 cells used for protein analysis were plated at low density onto 6‐well cell plates (Eppendorf 30720113) in culture medium and treated for each experimental condition at 50% confluence. RNA lysates were prepared using Norgen Biotek's Total RNA Purification Kit (Cat. 37500). Lysates were first treated with Promega RQ1 RNase‐Free DNase (Cat. M6101) and then converted to cDNA using Applied Biosystem's High‐Capacity RNA‐to‐cDNA Kit (Cat. 4387406). Quantitative real‐time PCR (qPCR) with SYBR Green (Bio‐Rad; SsoAdvanced Universal SYBR Green Supermix, Cat. 1725271) was carried out to assess gene expression. All results were normalized to ACTB. Primers for qPCR were ordered from Eton Bioscience. Primer sets used were as follows: ACTB‐Fwd 5'‐CACCATTGGCAATGAGCGGTTC‐3′, ACTB‐Rev 5'‐AGGTCTTTGCGGATGTCCACGT‐3′, p16‐Fwd 5'‐CCAACGCACCGAATAGTTAC‐3’, p16‐Rev 5'‐GCGCTGCCCATCATCATG‐3′.

## CONFLICT OF INTEREST

The authors declare that they have no conflict of interest.

## AUTHORS' CONTRIBUTIONS

P.R.C. conceptualized the project and all experiments, constructed the RPE p16‐mCherry reporter cell line, performed image analysis, created figures, and wrote the manuscript. P.R.C., S.K.G., and K.A.G. assisted in cell maintenance, western blot, and immunofluorescence experiments. M.A.R. assisted in cell maintenance and western blot experiments. S.R.S. assisted in cell maintenance and immunofluorescence experiments.

## Supporting information

Fig S1‐S3

## Data Availability

The data that support the findings of this study are openly available at https://doi.org/10.17632/m2ps5jpyzv.1.
