# Supplementary material for: Autophagy regulates the localization and degradation of p16INK4a
Source: Aging Cell. 2020 Jul 13;19(7):e13171. doi: 10.1111/acel.13171 (PMC7370706; doi:10.1111/acel.13171)
Supplement: Supplementary file 1 — Fig S1‐S3 [file ACEL-19-e13171-s001.pdf]

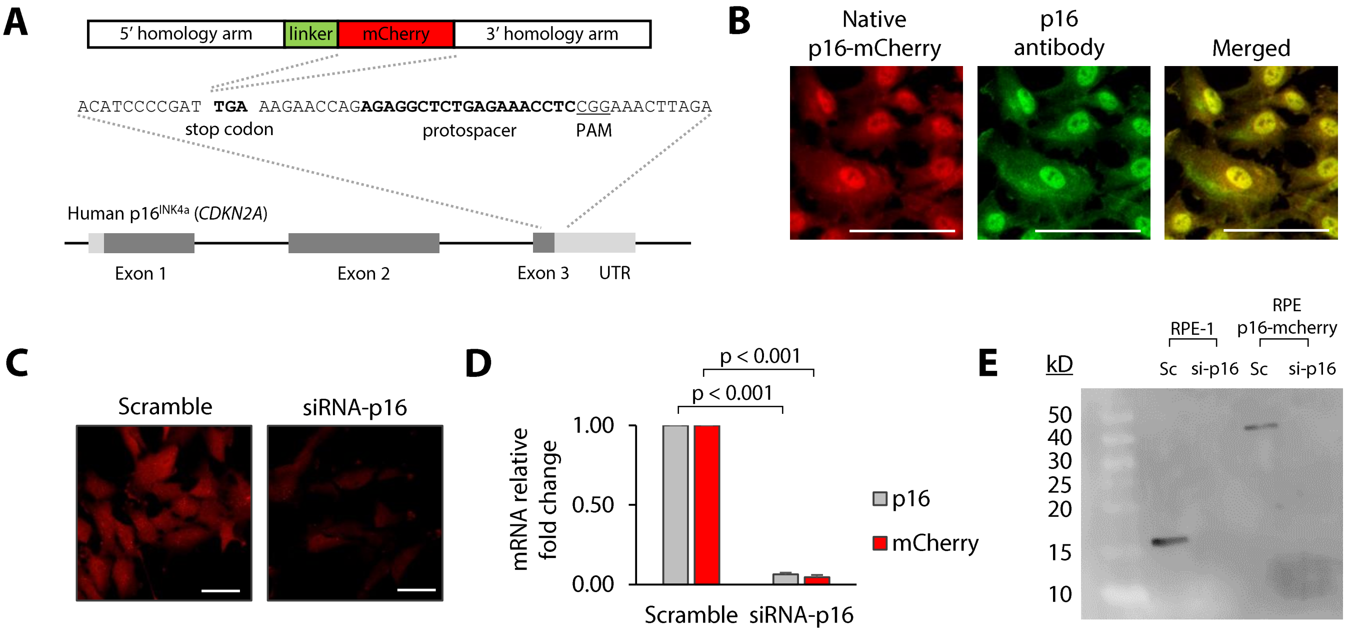

F

TGAAAGAACCAGAGAGGCTCTGAGAAACCTCCGGAAACTTAGATCATCAGTCACCGAAGGTCCTACAGGGCCACAACTGCCCCCGCCACAACCCACCCCG
CTTTCGTAGTTTTCATTTAGAAAATAGAGCTTTTAAAAATGTCCTGCCTTTTAACGTAGATATATGCCTTCCCCCACTACCGTAAATGTCCATTTATATC
ATTTTTTATATATTCTTATAAAAATGTAAAAAAGAAAAACACCGCTTCTGCCTTTTCACTGTGTTGGAGTTTTCTGGAGTGAGCACTCACGCCCTAAGCG
CACATTCATGTGGGCATTTCTTGCGAGCCTCGCAGCCTCCGGAAGCTGTCGACTTCATGACAAGCATTTTGTGAACTAGGGAAGCTCAGGGGGGTTACTG
GCTTCTCTTGAGTCACACTGCTAGCAAATGGCAGAACCAAAGCTCAAATAAAAATAAAATAATTTTCATTCATTCACTCATTTATTGTCAACATTTATTG
AGCACCTATTACAACAATTTCATCGCATGGAAGACAGCATCGTTTCTGACACTGTTGTTTCATGTATCTCTTAGAAAAACGCTGCTATTAGACATCTAAC
ACTATTTATCTTGAGGTGATAAAATATCAAAAGCCGTGTCTCAAGATCGATGAAATGCGGTTAAAATGATGAATAGAAACTCTAGGGGGACCTCATATCG
ATAGACTCGAGACTGGCACATCTGGAGATCCGTATTTATCCGGCTTCCCCTTCCAGATCACGCGAGGTTTGGGATATTTTGCTCACCAGGCCTCAGCCAG
GTAACTGAATCCAGCCAACCCTGGCCCATAGTCTCGGAATCCGACTCGGCTCCCAGTCCCCGCCTCGGCGTTCTGAGACCCCCAGGCTGGGGTTCCAGAG
GGCTGTGAGGTTGCGAATGACTGCTGCCAAACCGGAAGGAACTCTGCGGTTCTCTGCCACAGTGGGATTGTTGCAGGCACGCGGCTCAGACTTCACTGAG
GTTGGGAGATGCTCCTGTCCACGCTGCCTCATCCCGTGCTGGAGCACTGCACCTCTATTTTTTTTTTTTAGGGTACACGCCACATAA

G

GAGCGCGTGATGAACTTCGAGGACGGCGGCGTGGTGACCGTGACCCAGGACTCCTCCCTGCAGGACGGCGAGTTCATCTACAAGGTGAAGCTGCGCGGCA
CCAACTTCCCCTCCGACGGCCCCGTAATGCAGAAGAAGACCATGGGCTGGGAGGCCTCCTCCGAGCGGATGTACCCCGAGGACGGCGCCCTGAAGGGCGA
GATCAAGCAGAGGCTGAAGCTGAAGGACGGCGGCCACTACGACGCTGAGGTCAAGACCACCTACAAGGCCAAGAAGCCCGTGCAGCTGCCCGGCGCCTAC
AACGTCAACATCAAGTTGGACATCACCTCCCACAACGAGGACTACACCATCGTGGAACAGTACGAACGCGCCGAGGGCCGCCACTCCACCGGCGGCATGG
ACGAGCTGTACAAGTGAAAGAACCAGAGAGGCTCTGAGAAACCTCCGTAAACTTAGATCATCAGTCACCGAAGGTCCTACAGGGCCACAACTGCCCCCGC
CACAACCCACCCCGCTTTCGTAGTTTTCATTTAGAAAATAGAGCTTTTAAAAATGTCCTGCCTTTTAACGTAGATATATGCCTTCCCCCACTACCGTAAA
TGTCCATTTATATCATTTTTTATATATTCTTATAAAAATGTAAAAAAGAAAAACACCGCTTCTGCCTTTTCACTGTGTTGGAGTTTTCTGGAGTGAGCAC
TCACGCCCTAAGCGCACATTCATGTGGGCATTTCTTGCGAGCCTCGCAGCCTCCGGAAGCTGTCGACTTCATGACAAGCATTTTGTGAACTAGGGAAGCT
CAGGGGGGTTACCGGCTTCTCCTGAGTCACACTGCTAGCAAATGGCAGAACCAAAGCTCAAATAAAAATAAAATAATTTTCATTCATTCACTCATTTATT
GTCAACATTTATTGAGCACCTATTACAACAATTTCATCGCATGGAAGACAGCATCGTTTTTGACACTGTTGTTTCATGTATCTCTTAGAAAAACGCTGCT
ATTAGACATTTAACCCTATTTATCTTGAGGTGATAAAATATCAAAAGCCGTGTTTCAAGATCGATGAAATGCGGTTAAAATGATGAATAGAAACTTTAGG
GGGACCTCATATCGATAGACTCGAGACTGGCCCATTTGGAGATCCGTATTTATCCGGCTTCCCCTTCCAGATCCCGCGAGGTTTGGGATATTTTGCTCCC
CAGGCCTCAGCCAGGTAACTGAATCCAGCCAACCCTGGCCCATAGTTTCGGAATCCGACTCGGCTCCCAGTCCCCCCCTCGGCGTTTTGAGACCCCCCGG
CTGGGGTTCCCGAGGGCTGGGAGGTTGCGAATGACTGCTGCCAAACCGGAAGGAACTTTGCGGTTTTTTGCCCCAGTGGGATTGTTGCAGGCCCGCGGCT
CAGACTTCCCTGAGGTTGGGAGATGCTCCCGTCCCCGCTGCCTCATCCCGTGCTGGAGCCCTGCCCCTTTATTTTTTTTTTTTAGGGTACACGCCACATAA

Supplemental figure 1. Development of a human RPE p16-mCherry reporter cell line. A) Schematic for CRISPR-mediated incorporation of a (Gly/Ser)12 linker and mCherry fluorescent protein at the p16/CDKN2A stop codon of RPE-1 cells. **B)** Immunofluorescence staining of fixed RPE p16-mCherry cells. Native p16-mCherry in red. p16-antibody in green. Scale bars = 100 µm. **C)** Live RPE p16-mCherry cells transfected with siRNA-p16 or scramble control for 24 hours. Scale bars = 100 µm. **D)** RT-qPCR of siRNA-transfected cells with primers for p16 or mCherry. Transcripts normalized to RPS9 and fold-change relative to respective scramble control are shown. Statistical significance determined by two-tailed t-test (n = 3). **E)** Western blot stained for p16 from RPE-1 and RPE p16-mCherry cells treated with siRNA targeting p16 (si-p16) or scramble control (Sc). Predicted p16 molecular weight = 16 kD. Predicted p16-mCherry molecular weight = 44 kD. **F)** Sequencing near p16 stop codon of parental RPE-1 line used to create RPE p16-mCherry line. Stop codon and sequence used for 3′-homology arm underlined. **G)** Sequencing near mCherry-p16 stop codon of RPE p16-mCherry line. mCherry bolded. Stop codon and homology arm incorporated from donor plasmid underlined.

Supplemental Figure 2

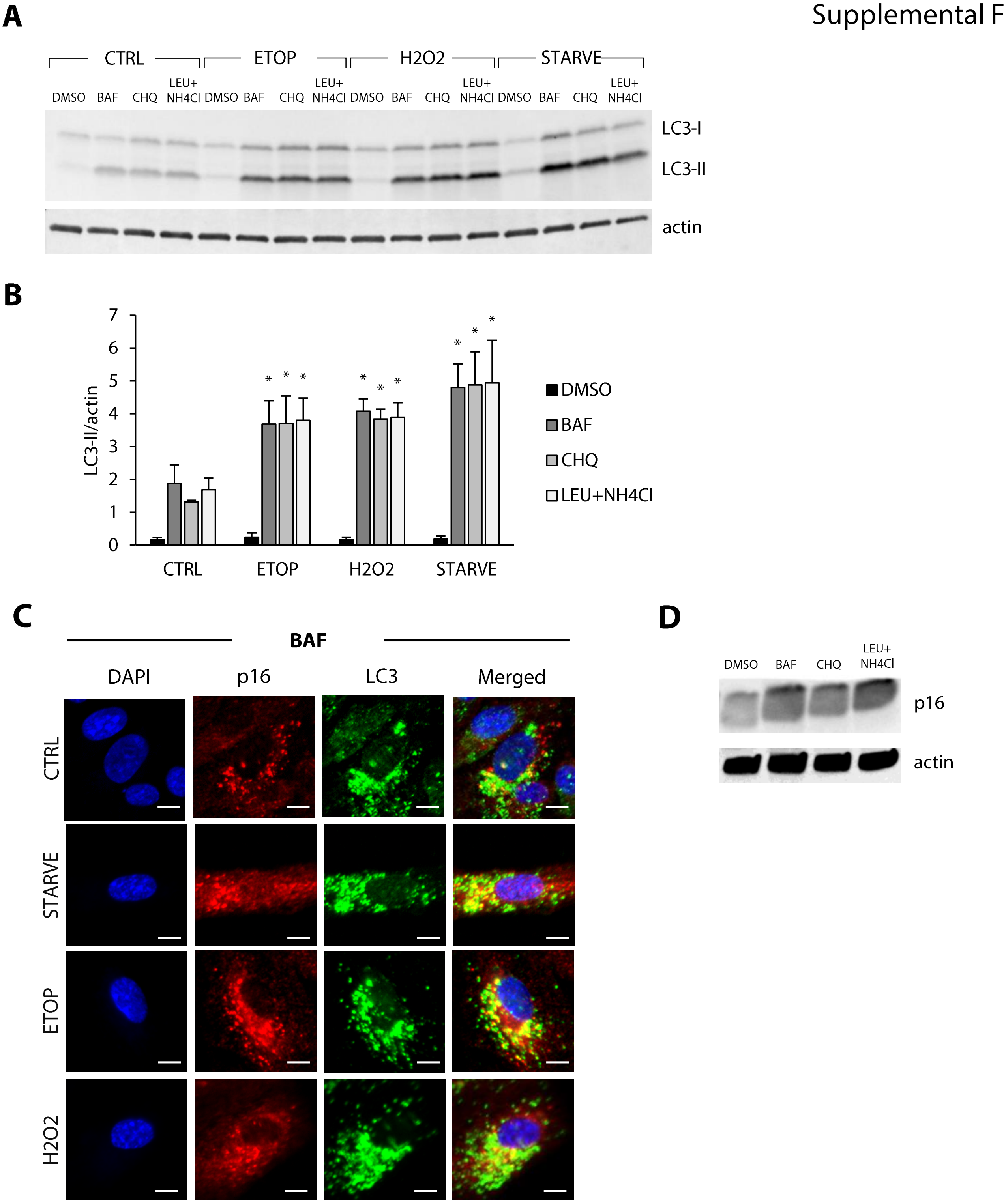

Supplemental figure 2. RPE-1 cells were treated with etoposide (20 μM), H2O2 (200 μM), or serum starved for 24 hours. Additionally, each group was treated with DMSO, bafilomycin (100 nM), chloroquine (40 μM), or leupeptin (100 μM) with NH4Cl (10 mM). Cells were then lysed for whole-cell protein analysis via western blot. **A)** Representative western blot. **B)** Quantification of western blot showing quantified LC3-II/actin. Statistical significance determined using two-way ANOVA (n = 3). * = $p<0.05$ relative to respective CTRL. All error bars = standard deviation. **C)** Representative immunofluorescence images for Figure 4C-4E for cells treated with bafilomycin (100 nM) for 24 hours. DAPI shown in blue; p16 shown in red; LC3 shown in green. Scale bars = 10 μm. **D)** Representative western blot for Figure 3F and Figure 4F. Cells were treated with DMSO, bafilomycin (100 nM), chloroquine (40 μM), or leupeptin (100 μM) with NH4Cl (10 mM) for 24 hours. Cells were then lysed for whole-cell protein analysis via western blot.

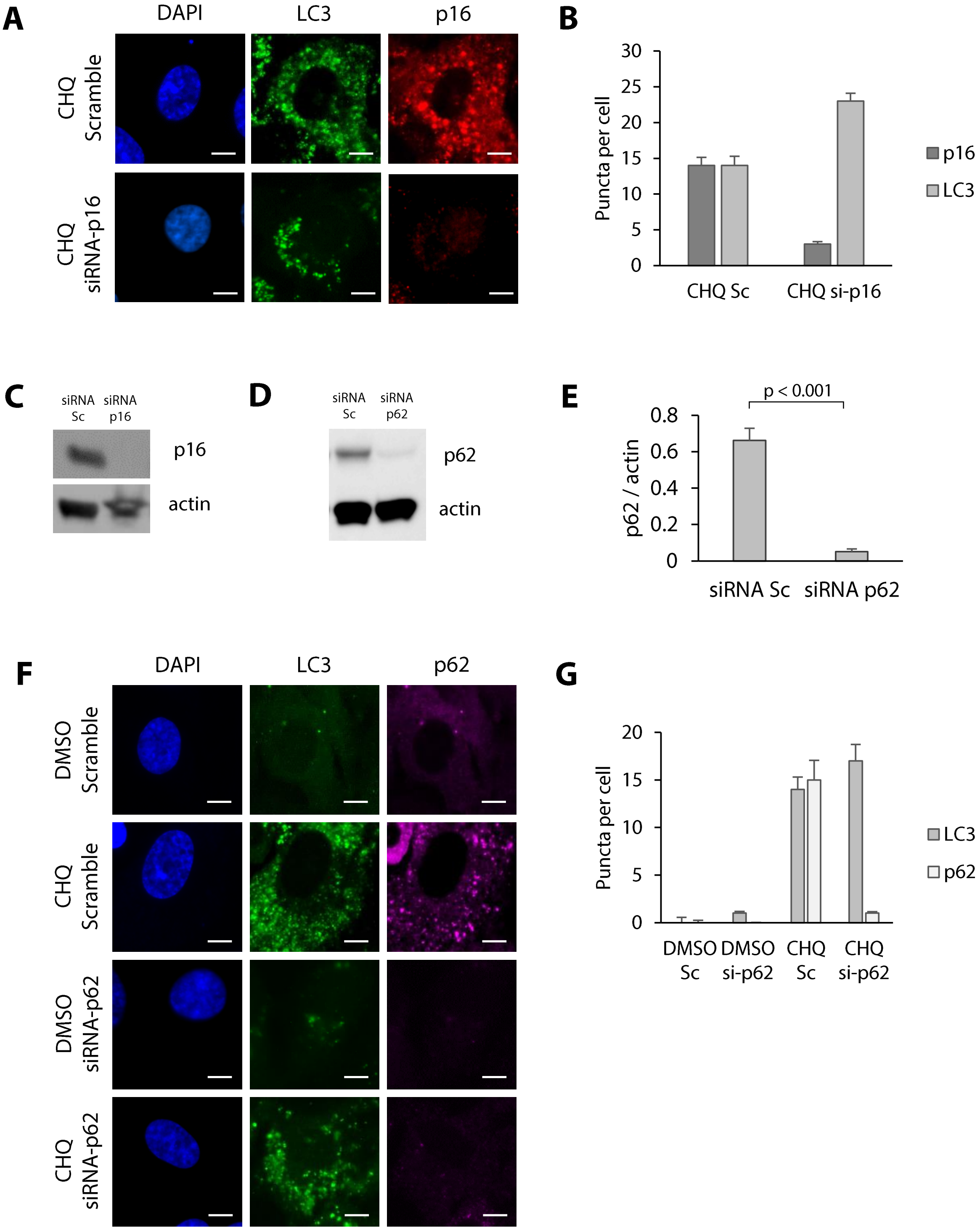

Supplemental figure 3. A) RPE-1 cells were treated with 40 μM chloroquine to block autophagy and transfected with siRNA-p16 (si-p16) or siRNA-scramble control (Sc) for 24 hours. Cells were then fixed, permeabilized with digitonin, and stained for DAPI (blue), p16 (red) and LC3 (green). Scale bars = 10 μm. **B)** Quantification of puncta segmented per cell for p16 knockdown experiment. **C)** Western blot for p16 knockdown via siRNA. **D)** Representative western blot for p62 knockdown via siRNA. **E)** Quantification of p62 knockdown western blot. Statistical significance performed using two-tailed student's t-test. n = 3. **F)** RPE-1 cells were treated with DMSO or 40 μM chloroquine to block autophagy and transfected with siRNA-p62 (si-p62) or siRNA-scramble control (Sc) for 24 hours. Cells were then fixed, permeabilized with digitonin, and stained for DAPI (blue), LC3 (green), and p62 (magenta). Scale bars = 10 μm. **G)** Quantification of puncta per cell from immunofluorescence for p62 knockdown experiment. All error bars = standard error of the mean.
